# Elsinochrome phytotoxin production and pathogenicity of *Elsinoë arachidis* isolates in China

**DOI:** 10.1371/journal.pone.0218391

**Published:** 2019-06-13

**Authors:** WenLi Jiao, Lu Liu, RuJun Zhou, MengXue Xu, Di Xiao, Caiyun Xue

**Affiliations:** Department of Plant Pathology, College of Plant Protection, Shenyang Agricultural University, Shenyang, Liaoning, China; Fujian Agriculture and Forestry University, CHINA

## Abstract

Peanut scab caused by *Elsinoë arachidis* is found throughout China’s peanut-growing areas. Elsinochrome produced by *E*. *arachidis* is a perylenequinone photosensitive mycotoxin vital to the pathogenic process of the pathogen. In this study, the complex mechanism underlying the regulation of elsinochrome biosynthesis by *E*. *arachidis* was investigated based on various nutritional and environmental factors. The initiation of elsinochrome biosynthesis depends on light. *E*. *arachidis* produced substantially more quantities of elsinochrome when grown on a semi-synthetic medium (PDA) than when grown on synthetic media with defined ingredients in the presence of light. Elsinochrome accumulation decreased when adjusted with either citrate or phosphate buffers and changing pH suppressed the radical growth. At temperatures ranging from 10°C to 25°C, the production of elsinochrome increased, peaking at 28°C, and it decreased slightly at 30°C. 63 field-collected isolates from China were assessed for the level of elsinochrome production, and pathogenicity analysis was conducted by selecting 12 strains from each 3 of the 4 groups with different levels of elsinochrome production. A direct correlation was observed between elsinochrome production and pathogenicity among the isolates. The results showed elsinochrome biosynthesis to be controlled by *E*. *arachidis* and showed elsinochrome to be a vital virulence factor of *E*. *arachidis*, required for disease severity.

## Introduction

Peanut scab disease is a widespread fungal disease, of considerable economic importance in China’s peanut-producing areas. The pathogen, *Elsinoë arachidis* (Bitanc. & Jenkins) Rossman & W.C. Allen (anamorph: *Sphaceloma arachidis* Bitanc. & Jenkins), infects stalks, petioles, and leaf blades, causing yield losses of 10–30% in susceptible peanut cultivars by stunting leaves or defoliation [1–2]. Disease symptoms are easy to recognize, given the cork-like and scab-like appearance of older infected tissues [3].

Elsinochrome appears red or orange in color in the medium, produced by *E*. *arachidis* and many other species of the phytopathogenic agent *Elsinoë* [4–7]. It is a virtually light-activated and nonhost-selective phytotoxin, sharing the same 4,9-dihydroxy-3,10-perylenequinone chromophore with cercosporin (*Cercospora* spp.), altertoxin I (*Alternaria alternata*), and phleichrome (*Cladosporium* spp.) whose structures fit perylenequinone toxins [8–11]. It has commonly assumed that these toxins can absorb light energy and convert to a triplet state in which they are more energetically activated and generate reactive oxygen species, which called photosensitizers[12].

Elsinochrome has been reported to induce necrotic lesions on citrus leaves, causing electrolyte leakage from citrus cells and toxicity to tobacco cells [13]. This effect is primarily attributed to the high yield of singlet oxygen and superoxide. Disrupted *Efpks1* in *E*. *fawcettii* completely abolished the production of elsinochrome, and the ability to develop lesions on citrus was significantly reduced, suggesting that elsinochrome produced by *E*. *fawcettii* fungi at full virulence [12–14].

Secondary metabolite biosynthesis by microbes can respond to environmental and nutritional factors [15]. Understanding the biosynthetic pathway of secondary metabolite toxins can help researchers study the role they play in disease [16]. According to previous studies, the accumulation of cercosporin was affected by complex factors, during which light serves as critical condition [17]. The production of elsinochrome by *E*. *fawcettii* has been proven to rely on light and pH as vital factors. However, the regulation of elsinochrome production by *E*. *arachidis* in response to environmental conditions and the diversity of elsinochrome production and fungal virulence by different *E*. *arachidis* isolates in China remains unclear.

For this reason, the objective of the present study was to investigate the effect of media, temperature, light, pH and cultivation time on biosynthesis of elsinochrome and growth of *E*. *arachidis* to determine whether isolates from different China’s peanut-producing areas produce elsinochrome and determine the relationship between this ability and fungal virulence.

## Materials and methods

### Strain and culture condition

*E*. *arachidis* strains used in this study were isolated from different peanut-growing areas in China (S1 Table). All strains were sub-cultured for purification by single spore and cultured on PDA under continuous light condition (5 microeinstein (μE) m^-2^s^-1^). For the preparation of fungal inoculum, 10-day-old mycelium was suspended in sterile water, and then the concentration was adjusted (OD_600_ = 2.0) (Zhao et al. 2017). Mycelium suspension (3ul) was placed on the surface of PDA (15ml) plate (90mm diameter). The colony diameter was measured after 4 weeks[5].

### Toxin extraction and quantitative analysis

Plates were incubated at 25°C under continuous fluorescent light. Colony diameters after inoculation were measured at 5, 10, 15, 20, 25, 30 and 35days in two perpendicular cross sections, respectively. For elsinochrome quantification, the method of Liao was referenced with slight modification [13]. 20 agar plugs (5mm diameter) were cut and extracted twice with acetone in the dark. Subsequently, the absorbance was measured at 468nm with spectrophotometer. Elsinochrome concentration was calculated using the standard curve method [5]. There were 3 replicate plates for each treatment.

### Culture conditions

To examine the effects of different media on fungal growth and elsinochrome production, various media included: PDA containing 200g potato, 15g dextrose and 15g agar; PSA replaced glucose with sucrose of PDA; complete medium (CM) containing 0.2g KH_2_PO_4_, 0.25g MgSO_4_·7H_2_O, 0.15 g NaCl, 1g Ca(NO_3_)_2_·4H_2_O, 10g glucose, 1g yeast extract, 1g casein hydrolysate, 15g agar; minimal medium (MM) except for yeast extract and casein hydrolysate of CM; malt extract agar (MEA) containing malt extract 20g, glucose 20g, agar 15g, peptone 1g; V8 agar (V8) containing 200ml V8 juice, 3g CaCO_3_, 15g agar; oatmeal agar (OA) containing 200g oatmeal, 15g agar; PLA containing peanut leaves 20g, glucose 20g, agar 15g [5]. The pH(3.1–7.6) of the medium was adjusted by the combination of citrate and phosphate buffers. The effects of temperature on fungal growth and elsinochrome production were assessed at 8 temperatures, namely 5, 10, 15, 20, 25, 28, 30, and 35°C. To determine the effect of light, the culture was incubated under 24h-light, 24h-dark and 12h photoperiod. There were 3 replicate plates for each treatment.

### Pathogenicity test

Pathogenicity of *E*. *arachidis* isolates was tested by inoculating mycelium suspension using the method of Zhao [18]. Baisha1016 susceptible to *E*. *arachidis* served as a host [1]. Mycelium suspension was sprayed onto the peanut leaves, and then incubated in a chamber under constant light condition at 25°C for lesion formation [19]. The disease severity was calculated by Fang with some modification[2]: grade 0 [0.0], no necrotic lesion; grade I [0.1], there are a few lesions and the leaves can develop normally; grade II [0.2], one fifth lesions and the leaves are slightly shrunk; grade III [0.4], the lesions took up nearly about one-third of the leaf area or the lesions on stem segment was connected to a strip; grade IV [0.6] leaves showed deformed wrinkles, petiole twisted, lesions took up nearly one-half of the leaf area; or three stem segments were densely healed; grade V [0.8] plants were significantly dwarfed, the parietal leaves were dead, or the leaves were scorched; or most of the stem segments were corked and rough; grade VI [1.0] branches die.

Disease index = (0.1n + 0.2n + 0.4n + 0.6n + 0.8n + 1.0n) / N ×100

n denotes the number of branches at each level; N is the total number of branches investigated

## Results

### Effect of environmental factors on growth and accumulation of elsinochrome

**Fig 1:**
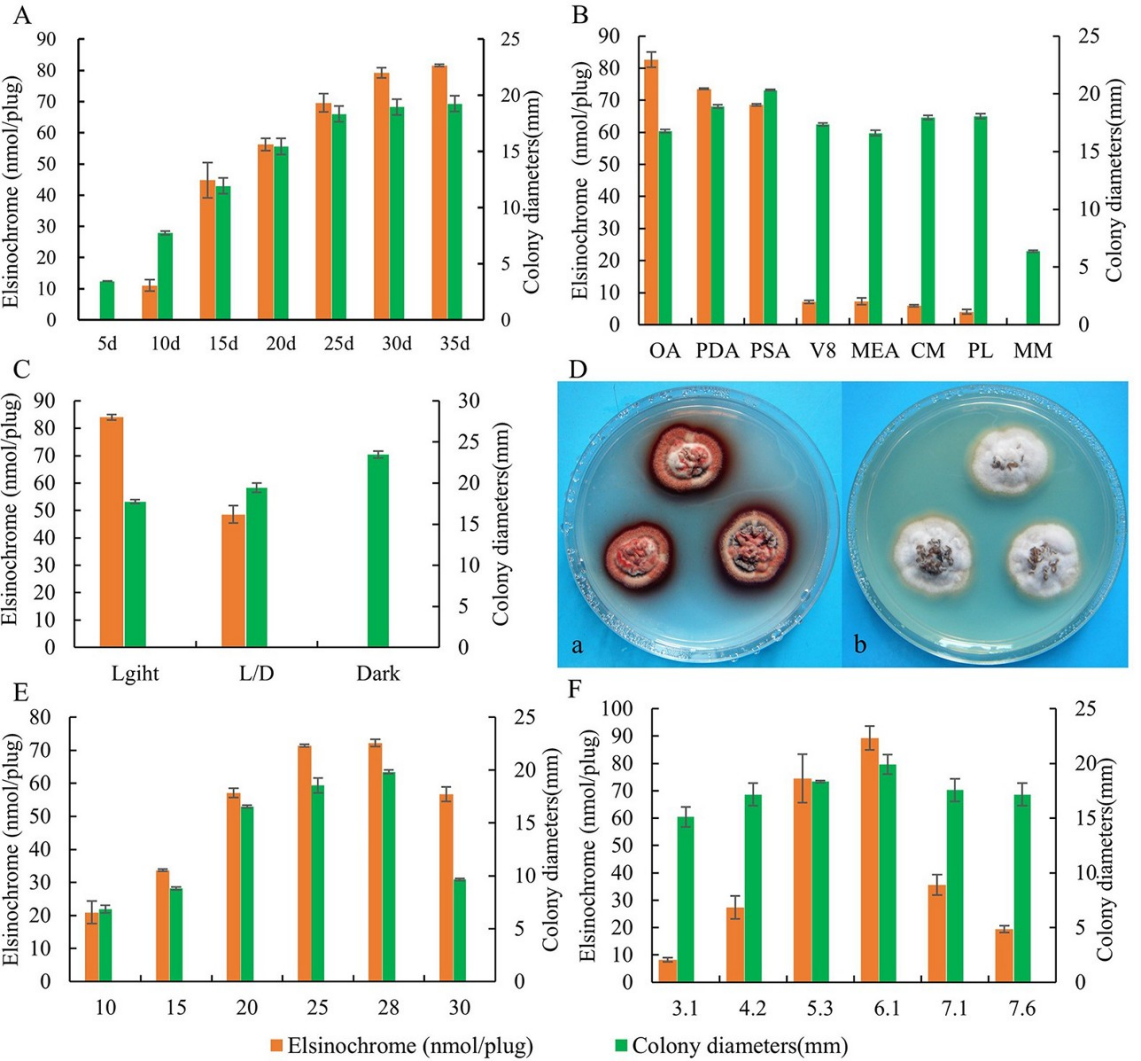
Effect of environmental factors on mycelial growth (mm) and the production of elsinochrome by isolate LNJH-C01 of *E*. *arachidis*. Accumulated dynamics of mycelial growth (mm) and the production of elsinochrome on PDA under 25°C constant light (A). Mycelial growth and the production of elsinochrome by isolate LNJH-C01 on PDA, PSA, OA, V8, MEA, MM, PL and CM(B); in constant light, 12h light-12h dark cycle (L/D), dark (C); in different temperature(E); in different pH(F). Colony morphology of LNJH-C01in different light condition. a: constant Light; b: darkness(D).

The radial growth rate of the *E*. *arachidis* was slow over the first 5d. Yet the growth rate increased rapidly in the subsequent 10-20d, colony diameter reached a maximum at 25d. The accumulation of elsinochrome generally increased as culture time continued, and then stabilized at 30d. Therefore, maximum toxin production was detected at 30d (Fig 1A).

Growing isolate on OA, PDA, and PSA led to precocious elsinochrome production in the presence of light, however, the accumulation of elsinochrome decreased 10-fold when the isolate was grown on CM. Interestingly, the production of elsinochrome was undetectable on MM, the composition of MM was the same as that of CM, only yeast extract and casein hydrolysate. MM cannot support fungal growth and produce less elsinochrome in MM than CM, suggesting that casein hydrolysate and yeast extract can support fungal radial growth and maximal elsinochrome production. The largest elsinochrome production was found on OA, and PSA was considered the optimum growth medium (Fig 1B).

Mycelial growth and elsinochrome production of *E*. *arachidis* isolate were significantly affected by light. Elsinochrome production was greatest in the presence of light, and decreased sharply when *E*. *arachidis* was placed under 12h photoperiod conditions, completely absent in the dark. In addition, dark conditions were most suitable for colony growth (Fig 1C).

Colony growth increased as the temperature rose from 10°C to 25°C. It peaked at 28°C and was slightly and rapidly diminished at 30°C. It did not grow at 5°C or 35°C. The range of temperature 25–28°C contributed to elsinochrome production (Fig 1E).

The fungus can grow within the pH range of 3.1–7.6 with citrate or phosphate buffers. The optimum pH was found in unbuffered PDA. Colony diameter of the isolate and elsinochrome accumulation both decreased when the fungus was grown on alkaline or acidic medium (Fig 1F).

### Accumulation of elsinochrome of 63 isolates of *E*. *arachidis* in culture

**Fig 2:**
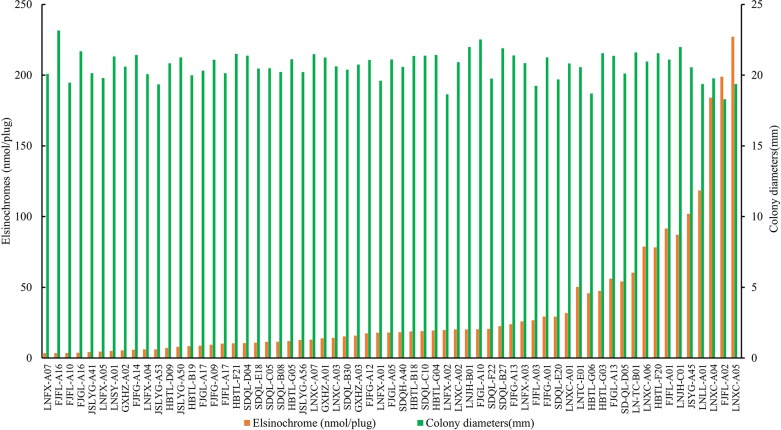
Elsinochrome accumulation and colony growth of 63 isolates of *E*. *arachidis*. Strains were incubated at 25°C on PDA under continuous fluorescent light. Colony diameters were measured at 30d after inoculation, as for elsinochrome production, 20 agar plugs (5-mm diameter) were cut and extracted twice with acetone and the absorbance was measured at 468 nm under a spectrophotometer. 3 replicate plates for each treatment.

Most *E*. *arachidis* isolates collected from China produce red pigments in culture. A wide range (3.45–227.11 nmol·plug^-1^) of elsinochrome was quantified (Fig 2). The 63 isolates could placed in 4 groups according to the production of elsinochrome by SPSS clustering, the average toxin production of each group was 14.04±7.71 nmol·plug^-1^ (48 isolates), 52.36±5.50 nmol·plug^-1^ (6 isolates), 92.66±15.41 nmol·plug^-1^ (6 isolates), and 203.37±21.85 nmol·plug^-1^ (3 isolates), respectively. Pearson correlation coefficient was used to analyze the relationship between colony growth and elsinochrome accumulation. r = -0.297, *P* = 0.018, indicating a negative correlation between the accumulation of elsinochrome and colony growth.

### Pathogenicity test

**Fig 3:**
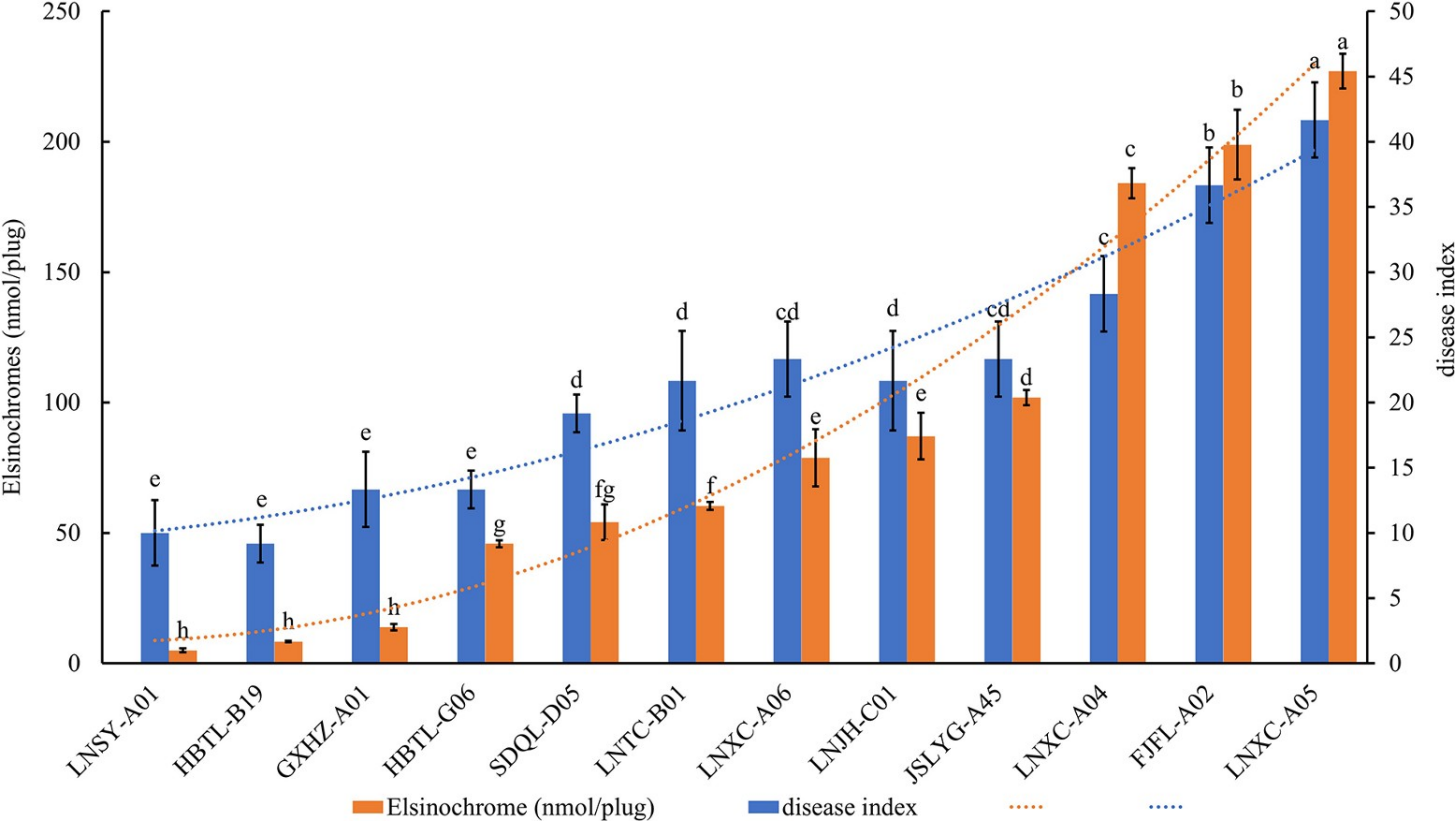
Pearson correlation analysis among disease index and elsinochrome. Pathogenicity of *E*. *arachidis* isolates was tested by inoculating mycelium suspension. The correlation between disease index and the elsinochrome accumulation in culture were calculated by SPSS 19.0.

A total of 12 isolates cultured from 4 different elsinochrome groups were assessed for pathogenicity on peanut leaves and production of necrotic lesions. LNXC-A05 was highly virulent to peanut, LNSY-A01 exhibited reduced virulence, LNJH-C01 was moderately virulent, and the disease index was 41.7, 10.0, and 20.97, respectively (Fig 3). Examination of the correlation between disease index and the yield of elsinochrome accumulation in culture showed Pearson correlation coefficient r = 0.964, *P* = 4.196 × 10^−7^, suggesting a direct correspondence between the accumulation of elsinochrome and pathogenicity (Fig 3). As stated above, elsinochrome is considered a vital virulence factor of peanut scab.

## Discussion

*Elsinoë* species are common phytopathogens causing scab and spot on field crops (e.g. cassava, bean, peanut and ornamentals) and economic crops (e.g. poinsettias, avocado, mango, grape and citrus) [3]. Many *Elsinoë* can produce elsinochrome [6–7]. Elsinochrome is a light-activated, nonhost-selective phytotoxin that can damage cell membranes and induce electrolyte leakage. Recent studies based on molecular and genetic tools verified the critical role of elsinochrome in lesion development [20]. The ability to produce elsinochrome may serve be an important means by which *Elsinoë* can infect different crop species and cause disease.

The production and accumulation of elsinochrome showed stable increases over culture time under laboratory conditions, and these increases were affected by light, temperature, and pH. Here, light was found to be the most indispensable signal for elsinochrome biosynthesis in *E*. *arachidis*; elsinochrome production was completely abolished when the organism was kept in darkness. In *E*. *fawcettii*, although the biosynthesis of elsinochrome was suppressed in darkness, there was still a small amount of synthesis. The ambient pH was found to be one of the most important signals suitable for use as a regulatory cue for secondary metabolites and developmental processes in many organisms [21–22]. LNJH-C01 produces large quantities of elsinochrome when grown at PDA and it suppressed radical growth across different pH values in the presence of citrate and phosphate. However, the quantities of elsinochrome in *E*. *fawcettii* were largest when it was grown under alkaline conditions on PDA which indicated the complexity of metabolic pathway, even the same substance is synthesized differently in different species. In general, elsinochrome biosynthesis of *Elsinoë*. spp was complex and showed differences between species.

A wide range of levels of elsinochrome accumulation was observed among isolates of *E*. *arachidis* from different parts of China. In the previous study, elsinochrome, which induced necrotic lesions on peanut leaves, was demonstrated to be an important virulence factor to *E*. *arachidis* [4]. To assess the correlation between the production of elsinochrome and the pathogenicity of pathogens, we selected 12 strains for pathogenicity assays and grouped them by differences in toxin production. Pearson correlation coefficient was used to analyze the relationship between pathogenicity and elsinochrome accumulation, r = 0.964, suggesting that among *E*. *arachidis* isolates the accumulation of elsinochrome in PDA and the pathogenicity had a direct correspondence. However, the manner by which elsinochrome causes virulence and what the role elsinochrome may play in the mechanism underlying pathogenesis of *E*. *arachidis* needs further study.

In general, a complex, interrelated regulatory network causes to the accumulation of elsinochrome in *E*. *arachidis*. Direct correspondence between pathogenicity and elsinochrome accumulation here support our previous finding that elsinochrome is necessary to fungal virulence.

## Supporting information

S1 Raw data Mycelial growth and the production of elsinochrome of LNJH-C01 under different enviromantal conditions. (XLS)

S1 Table The information of 63 isolates of *E*. *arachidis*. (XLS)
